# Chemoradiotherapy vs radiotherapy for nonoperative early stage esophageal cancer: A seer data analysis

**DOI:** 10.1002/cam4.3132

**Published:** 2020-05-22

**Authors:** Jiaxin Li, Yibin Jia, Yufeng Cheng, Jianbo Wang

**Affiliations:** ^1^ Department of Radiation Oncology Qilu Hospital Cheeloo College of Medicine Shandong University Jinan Shandong China

**Keywords:** chemoradiotherapy (CRT), early stage, Esophageal cancer (EC), nonoperative, radiotherapy (RT)

## Abstract

**Background:**

The benefit of endoscopic treatment (ET) and esophagectomy for early esophageal cancer (EC) has been sufficiently recognized. Radiotherapy (RT) is the main treatment modality for patients who do not undergo surgery. The effectiveness of adding chemotherapy (CT) to RT remains unclear. This study aimed to evaluate the impact of chemoradiotherapy (CRT) and RT alone on overall survival (OS) and cancer‐specific survival (CSS) in early EC patients not undergoing surgery.

**Methods:**

Data collected between 2004 and 2015 were obtained from the national Surveillance, Epidemiology, and End Results (SEER) database. All the samples were randomly grouped into the training cohort or the verification cohort. The training cohort was split into subgroups by stage, age, and histology. Stage was based on the American Joint Committee on Cancer (AJCC) 6th edition published in 2004. The Kaplan‐Meier method and Cox proportional hazards modeling were used to compare OS and CSS. The performance of the nomogram was measured by a concordance index (C‐index) and the calibration curve.

**Results:**

Data for a total of 5332 patients were obtained from the SEER database. A total of 3736 patients (stage I: n = 1277; stage IIA: n = 1484; stage IIB: n = 975) were used for the training cohort. Multivariate Cox regression analysis showed that age, sex, histology, grade, therapy, reasons for no surgery, and year of diagnosis were independent predictors of OS. The survival curve of patients treated with CRT showed a significant survival benefit compared to that in patients treated by RT alone in stage I, stage IIA, and stage IIB. CRT was also found to be related to better survival than RT in patients at a younger age (<65) and an older age (≥65) with squamous cell carcinoma or adenocarcinoma.

**Conclusions:**

Compared with RT, CRT results in better OS and CSS in early EC patients who do not undergo surgery.

## Graphical abstract

Chemoradiotherapy results in a better OS and CSS in early esophageal cancer patients who do not undergo surgery.

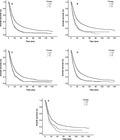

## 1 INTRODUCTION

Esophageal cancer (EC) is the eighth most common cancer worldwide and the sixth leading cause of cancer‐related mortality.^1^ Approximately 455 800 new cases and 400 200 deaths worldwide were diagnosed in 2012.^2^ In the US alone, the number of estimated new cases of EC was approximately 17 000, and the number of estimated deaths was more than 15 000 in 2016; nearly 80% of cancer‐related deaths occurred in males.^3^ Squamous carcinoma is still the most common subtype worldwide. However, the incidence of adenocarcinoma is increasing in Western countries.^4^ EC is mostly diagnosed at an advanced stage, and the standard treatment of those patients is widely accepted. However, for early stage EC, no consensus has been reached for the most effective treatment. An epidemiological investigation of EC^5^ stated that early stage cancer occurred in 22% of all EC patients diagnosed from 1998 to 2009. Patients in this large population are recommended endoscopic treatment (ET) or esophagectomy according to the National Comprehensive Cancer Network (NCCN) guidelines. For patients who refuse or are intolerant of surgery, experts have tried a variety of treatments. A retrospective study confirmed the feasibility and effectiveness of concurrent chemoradiotherapy (CRT) with low‐dose continuous infusion of 5‐fluorouracil and cisplatin for stage I‐II EC.^6^ A Japanese clinical study^7^ concluded that CRT for EC patients in stage I/II showed good prognosis. However, the stage I/II subgroup included only 25 patients. Early EC is a localized lesion, and we question whether the addition of systemic chemotherapy (CT) will bring benefits. There has been controversy over the appropriate treatment for nonoperative patients with early stage EC. To our knowledge, there is no comparison between radiotherapy (RT) and CRT for those patients.

Due to the lack of clinical trials, we used the Surveillance, Epidemiology, and End Results (SEER) database to access information. The aim of this study was to compare the appropriate therapy for nonoperative patients with early EC (stage I/II) by retrospective analysis of the SEER database.

## 2 MATERIALS AND METHODS

### 2.1 Patients

For this study, nonoperative EC patients treated with RT, CT, or CRT and whose disease was in an early stage (T_1‐3_N_0‐1_M_0_) were eligible; early stage included stage I, stage IIA, and stage IIB, according to the American Joint Committee on Cancer (AJCC) 6th edition published in 2004. The form of radiation was beam radiation, and CT was used as a synchronous or sequential step after RT. Patients with missing or unknown information were excluded. Data for a total of 5332 nonoperative EC patients from the period between 2004 and 2015 were derived from the SEER database. The analysis included age at diagnosis, sex, race, tumor site, histology, differentiation grade, stage, receipt of therapy, reasons for no surgery, year of diagnosis, cancer‐specific survival (CSS), and months of survival.

### 2.2 Statistical analysis

The study sample was randomly stratified into the training cohort and verification cohort (by R software 3.6.0). The training group accounted for 70% of the total sample and was used to establish a Cox proportional hazards model, ROC curve, nomogram, and survival curves. The verification cohort demonstrated accuracy. Multiple variables were age (<65, ≥65), sex, race (white, black, and other/unknown), tumor site (cervical, thoracic, and abdominal), histology, differentiation grade, stage, reasons for no surgery, year of diagnosis, and therapy. Multivariate Cox proportional hazard regression was used to determine independent predictors of mortality. *P* < .05 was considered statistically significant. ROC curves of 3‐year and 5‐year survival were used to verify the accuracy of the study. In the case of AUC ≥ 0.5, the closer the AUC is to 1, the better the diagnosis effect. A nomogram^8^ was generated from multivariate analysis aimed at predicting mortality. In the OS analysis, patient death was considered an event. In the CSS analysis, patients who survived or died of other causes were excluded, and death from EC was considered an event. Survival curves of the use of different therapies in stage I‐IIB, younger and older age, and squamous cell carcinoma and adenocarcinoma were evaluated by the Kaplan‐Meier method and compared with the log‐rank test. All analyses were performed using R software and IBM SPSS version 25.0.

This was a study using de‐identified data from the SEER database. Ethical approval by the ethical committee of the Qilu Hospital, Cheeloo College of Medicine, Shandong University was waived based on our institutional policy.

## 3 RESULTS

A total of 5332 nonoperative EC patients from the period between 2004 and 2015 were derived from the SEER database. The number in the training cohorts was 3736, accounting for 70% of the total. Table 1 includes all variables in a multivariate model to show demographics. As illustrated, older age (69.8%), male sex (71.7%), thoracic tumor site (85.9%), moderate‐poor differentiation (38.1%), and CT plus RT (74.7%) characteristics constituted a major part of the training cohort. The baseline characteristics were randomly distributed between the training and validation cohorts (*P* > .05). Table 2includes all variables in the multivariate model concerning OS and CSS. Younger age contributed to better OS than older age (HR = 0.878; 95%CI: 0.807‐0.955, *P* = .003). Features such as male sex (HR = 1.146; 95%CI: 1.050‐1.252; *P* = .002), poorly differentiated grade (HR = 1.333; 95%CI: 1.098‐1.618; *P* = .000), CT (HR = 1.659; 95%CI: 1.451‐1.897; *P* = .000), or RT (HR = 1.723; 95%CI: 1.563‐1.899; *P* = .000) were related to poor OS. As for the reasons for no surgery, patients were divided into three groups: those who were recommended surgery but denied it, those for whom surgery was not recommended due to contraindications, and those who had other unknown reasons for not undergoing surgery. Surgery‐recommended patients had a worse OS than other patients (HR = 0.833; 95%CI: 0.734‐0.944; *P* = .004). According to year of diagnosis (2004‐2015), there was a significant difference between the first 6 years (HR = 1.196; 95%CI: 1.107‐1.292; *P* = .000) and the last 6 years. Black, white (HR = 0.904; 95%CI: 0.806‐1.015; *P* = .087), and other races (HR = 0.842; 95%CI: 0.692‐1.026; *P* = .089) had similar OS. Cervical (HR = 0.945; 95%CI: 0.750‐1.189; *P* = .627) and thoracic tumor (HR = 0.978; 95%CI: 0.865‐1.106; *P* = .721) sites had OS similar to that of abdominal sites. No differences existed in squamous carcinoma, small cell cancer (HR = 0.976; 95% CI: 0.550‐1.732; *P* = .934), or adenocarcinoma (HR = 1.089; 95%CI: 0.998‐1.189; *P* = .056). In addition, stage I, stage IIA, or stage IIB had no impact on OS. Using data from patients whose deaths were directly related to this cancer to analyze CSS, white patients had relatively better CSS than patients of other races (HR = 0.839; 95%CI: 0.729‐0.964; *P* = .014). As expected, less differentiated tumors (moderately: HR = 1.325, 95%CI: 1.032‐1.700, *P* = .027; poorly: HR = 1.466, 95%CI: 1.142‐1.883, *P* = .003), stage IIB (HR = 1.223, 95%CI: 1.088‐1.376, *P* = .001), later year of diagnosis (HR = 1.250, 95%CI: 1.138‐1.373, *P* = .000), and other unknown causes of nonoperative status (HR = 1.769, 95%CI: 1.185‐2.640, *P* = .005) were related to poor CSS. Compared with squamous carcinoma, adenocarcinoma (HR = 1.268, 95%CI: 1.138‐1.413, *P* = .000) had a worse CSS. Similar CSS was found for age (HR = 1.100; 95%CI: 0.996‐1.214; *P* = .060) and tumor site (cervical: *P* = .107; thoracic: *P* = .226).

**Table 1:** Baseline characteristics of all patients and those in the training and validation cohorts

Characteristic	Total cohort	Training cohort	Alidation cohort	*P*
	n	n	n	
Age at diagnose (≥65 as ref.)	3721 (69.8%)	2607 (69.8%)	1114 (70.0%)	.331
<65	1611 (30.2%)	1129 (30.2%)	482 (30.0%)	
Race (black as ref.)	762 (14.3%)	528 (14.1%)	234 (14.7%)	.222
Other	304 (5.7%)	208 (5.6%)	96 (6.0%)	
White	4266 (80.0%)	3000 (80.3)	1266 (79.3%)	
Sex (female as ref.)	1483 (27.8%)	1058 (28.3%)	425 (26.6%)	.245
Male	3849 (72.2%)	2678 (71.7%)	1171 (73.4%)	
Site (abdominalas ref.)	546 (10.2%)	386 (10.3%)	160 (10.0%)	.218
Cervical	204 (3.8%)	140 (3.7%)	64 (4.0%)	
Thoracic	4582 (85.9%)	3210 (85.9%)	1372 (86.0%)	
Histology (squamous carcinoma as ref.)	2574 (48.3%)	1816 (48.6%)	758 (47.5%)	.281
Small cell cancer	25 (0.5%)	17 (0.4%)	8 (0.5%)	
Adenocarcinoma	2378 (44.6%)	1652 (44.2%)	726 (45.5%)	
Other	355 (6.6%)	251 (6.7%)	104(6.5%)	
Grade (grade I; Well diferentiated as ref.)	242 (4.5%)	179 (4.8%)	63 (3.9%)	.26
Grade II; Moderately diferentiated	2025 (38.0%)	1424 (38.1%)	601 (37.7%)	
Grade III; Poorly diferentiated	1978 (37.1%)	1383(37.0%)	595 (37.3%)	
Grade IV; Undiferentiated	64 (1.2%)	46 (1.2%)	18 (1.1%)	
Unknown	1023 (19.2%)	704 (18.8%)	319 (20.0%)	
Stage (stage I as ref.)	1828 (34.3%)	1277 (34.2%)	551 (34.5%)	.253
Stage IIA	2125 (40.0%)	1484 (39.7%)	641 (40.2%)	
Stage IIB	1379 (25.7%)	975 (26.1%)	404 (25.3%)	
Therapy (chemoradiotherapy as ref.)	4006 (75.2%)	2792 (74.7%)	1214 (76.1%)	.345
Chemotherapy	423 (7.9%)	301 (8.1%)	122 (7.6%)	
Radiotherapy	903 (16.9%)	643 (17.2%)	260 (16.3%)	
Reasonosurg (Not recommended as ref.)	4699 (88.1%)	3306 (88.5%)	1396 (87.5%)	.394
Recommended	579 (10.9%)	390 (10.4%)	189 (11.8%)	
Other	51 (1.0%)	40 (1.1%)	11 (0.7%)	
Dag year (2010‐2015 as ref.)	2904 (54.5%)	2042 (54.7%)	862 (54.0%)	.294
2004‐2009	2428 (45.5%)	1694 (45.3%)	734 (46.0%)	

Abbreviations: HR, hazard ratio; CI, confidence interval.

**Table 2:** Multivariate analysis of training cohort

Characteristic	OS		CSS	
	HR (95%C I)	*P*	HR (95%C I)	*P*
Age at diagnose (≥65 as ref.)				
<65	0.878 (0.807‐0.955)	.003	1.100 (0.996‐1.214)	.06
Race (black as ref.)				
Other	0.842 (0.692‐1.026)	.089	0.881 (0.699‐1.111)	.284
White	0.904 (0.806‐1.015)	.087	0.839 (0.729‐0.964)	.014
Sex (female as ref.)				
Male	1.146 (1.050‐1.252)	.002	1.055 (0.948‐1.174)	.324
Site (abdominalas ref.)				
Cervical	0.945 (0.750‐1.189)	.627	0.782 (0.581‐1.055)	.107
Thoracic	0.978 (0.865‐1.106)	.721	0.913 (0.789‐1.058)	.226
Histology (squamous carcinoma as ref.)				
Small cell cancer	0.976 (0.550‐1.732)	.934	1.161 (0.605‐2.229)	.654
Adenocarcinoma	1.089 (0.998‐1.189)	.056	1.268 (1.138‐1.413)	0
Other	1.342 (1.148‐1.569)	0	1.581 (1.311‐1.908)	0
Grade (grade I; Well diferentiated as ref.)				
Grade II; Moderately diferentiated	1.193 (0.983‐1.446)	.074	1.325 (1.032‐1.700)	.027
Grade III; Poorly diferentiated	1.333 (1.098‐1.618)	.004	1.466 (1.142‐1.883)	.003
Grade IV; Undiferentiated	1.222 (0.834‐1.791)	.303	1.433 (0.908‐2.263)	.123
Unknown	1.108 (0.904‐1.359)	.323	1.188 (0.914‐1.545)	.197
Stage (stage I as ref.)				
Stage IIA	0.978 (0.895‐1.069)	.624	1.024 (0.918‐1.144)	.668
Stage IIB	1.086 (0.985‐1.197)	.097	1.223 (1.088‐1.376)	.001
Therapy (chemo radiotherapy as ref.)				
Chemotherapy	1.659 (1.451‐1.897)	0	1.382 (1.163‐1.641)	0
Radiotherapy	1.723 (1.563‐1.899)	0	1.638 (1.452‐1.848)	0
Reasonosurg (Not recommended as ref.)				
Recommended	0.833 (0.734‐0.944)	.004	0.881 (0.759‐1.021)	.092
Other	1.452 (1.003‐2.102)	.048	1.769 (1.185‐2.640)	.005
Dag year (2010‐2015 as ref.)				
2004‐2009	1.196 (1.107‐1.292)	0	1.250 (1.138‐1.373)	0

Table 3 shows the age and histology subgroup analysis of the three kinds of treatment. Younger age (RT: HR = 1.518, 95%CI: 1.224‐1.883, *P* = .000; CT: HR = 1.769, 95%CI: 1.402‐2.233, *P* = .000) as well as older age (RT: HR = 1.587, 95%CI: 1.373‐1.835, *P* = .000; CT: HR = 1.575, 95%CI: 1.302‐1.904, *P* = .000) had poorer OS with CRT than with CT or RT alone. Squamous carcinoma (RT: HR = 1.954, 95%CI: 1.704‐2.240, *P* = .000; CT: HR = 1.883, 95%CI: 1.531‐2.316, *P* = .000) and adenocarcinoma (RT: HR = 1.587, 95%CI: 1.373‐1.835, *P* = .000; CT: HR = 1.575, 95%CI: 1.302‐1.904, *P* = .000) also showed the same trend. Table 4 shows CRT still confers a survival advantage in stage I (RT: HR = 1.699, 95%CI: 1.466‐1.968, *P* = .000; CT: HR = 1.860, 95%CI: 1.530‐2.260, *P* = .000), stage IIA (RT: HR = 1.817, 95%CI: 1.552‐2.128, *P* = .000; CT: HR = 1.824, 95%CI: 1.386‐2.400, *P* = .000), and stage IIB patients (RT: HR = 1.861, 95%CI: 1.499‐2.310, *P* = .000; CT: HR = 1.494, 95%CI: 1.168‐1.912, *P* = .001).

**Table 3:** Age and histology subgroups analysis of therapy

		≥65			<65	
	n	H R(95%CI)	*P*	n	H R(95%CI)	*P*
Therapy (chemoradiotherapy as ref.)	1879			913		
Chemotherapy	204	1.744 (1.485‐2.048)	0	97	1.769 (1.402‐2.233)	0
Radiotherapy	524	1.794 (1.611‐1.998)	0	119	1.518 (1.224‐1.883)	0

		Squamous carcinoma			Adenocarcinoma	
	n	H R(95%CI)	*P*	n	H R(95%CI)	*P*
Therapy (chemoradiotherapy as ref.)	1378		0	1221		0
Chemotherapy	121	1.883(1.531‐2.316)	0	153	1.575(1.302‐1.904)	0
Radiotherapy	317	1.954(1.704‐2,240)	0	278	1.587(1.373‐1.835)	0

**Table 4:** Different stage subgroups analysis of therapy

		Stage I	
	n	H R(95%CI)	*P*
Therapy (chemoradiotherapy as ref.)	840		
Chemotherapy	144	1.860 (1.530‐2.260)	.000
Radiotherapy	293	1.699 (1.466‐1.968)	.000

		Stage I IA	
	n	H R(95%C I)	*P*
Therapy (chemoradiotherapy as ref.)	1180		
Chemotherapy	69	1.824 (1.386‐2.400)	.000
Radiotherapy	236	1.817 (1.552‐2.128)	.000

		Stage II B	
	n	H R(95%C I)	*P*
Therapy (chemoradiotherapy as ref.)	772		
Chemotherapy	88	1.494 (1.168‐1.912)	.001
Radiotherapy	114	1.861 (1.499‐2.310)	.000

**FIGURE 1:**
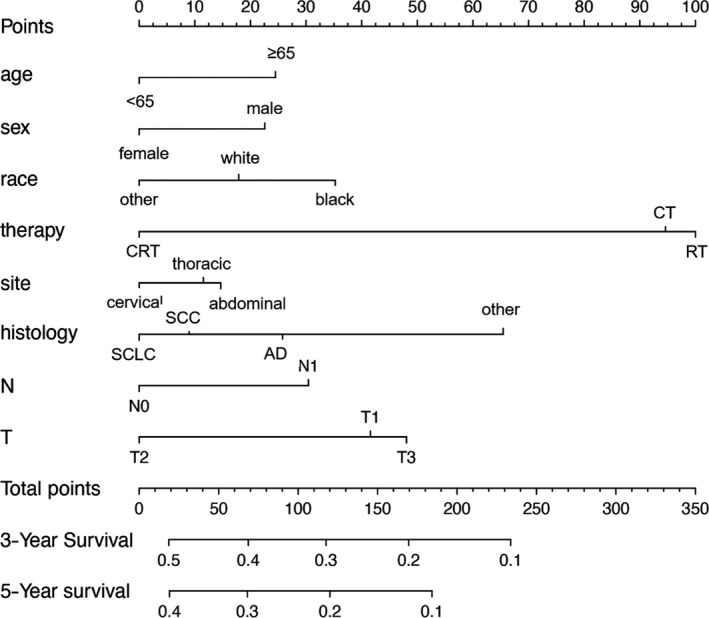
Nomogram predicting 3‐ and 5‐y survival for non‐operative esophageal cancer. AD, adenocarcinoma; SCC, squamous cell carcinoma; SCLC, small cell lung cancer

**FIGURE 2:**
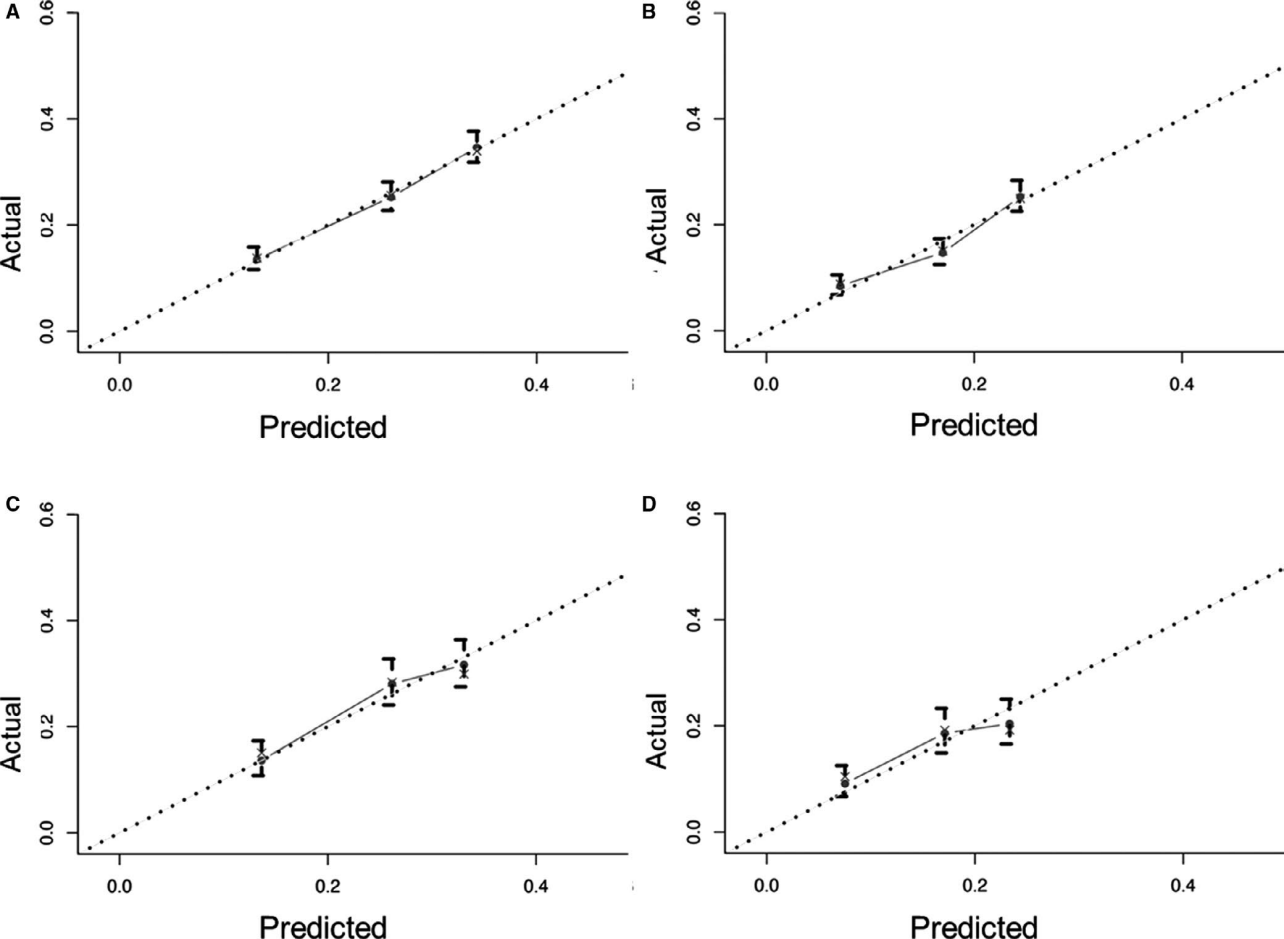
The calibration curves for predicting survival of 3‐ (A) and 5‐year(B) in the training cohort, and of 3‐(C) and 5‐y (D) in the verification cohort. Nomogram‐predicted survival is plotted on the *x*‐axis; actual survival is plotted on the *y*‐axis.

Nomograms are predictive models of the 3‐year and 5‐year survival of patients. As shown in Figure 1, tumor therapy contributes most to the prognosis, followed by histology, T stage, race, N stage, age, sex, and site. Every predictor corresponds to one score. All scores were added to predict a 3‐ to 5‐year survival rate. In other words, if the nomogram is tenable and can predict common patterns, an Asian female below 65 suffering from cervical squamous EC in T2N0M0 (stage IIA) who receives RT plus CT could acquire the highest survival rate in theory. The C‐index of the training cohort was 0.595 and that of the verification cohort was 0.587. The verification curves of the 3‐ and 5‐year survival rates show consistency between the predicted and observed situations (Figure 2).

**FIGURE 3:**
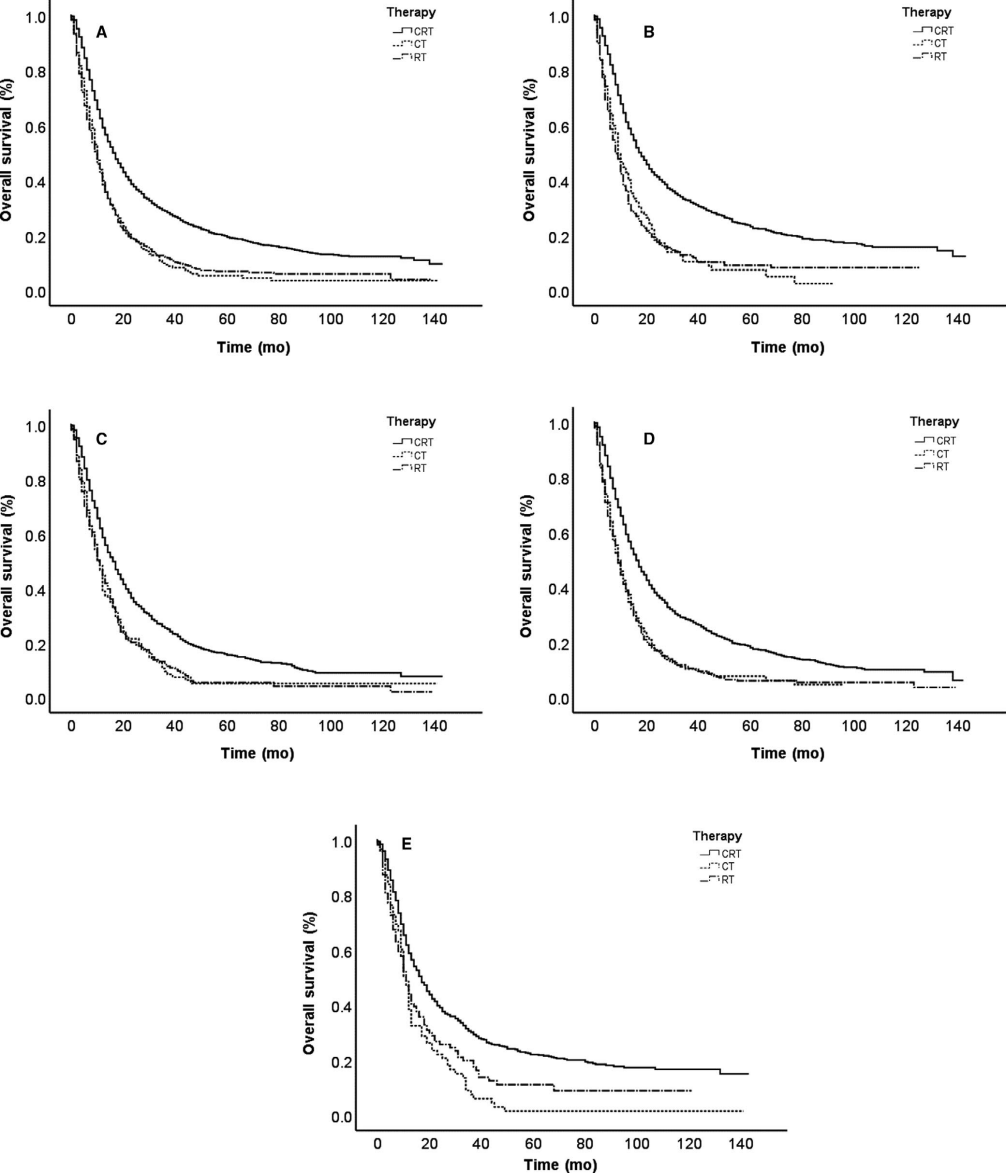
Overall survival of CRT, RT, CT for the total sample(A), squamous carcinoma(B), adenocarcinoma(C), ≥65(D) and <65(E).

The OS curves are shown in Figure 3 based on different cohorts. As presented in the total sample, no significant difference exists in RT and CT survival curves. RT and CT survival curves tend to be in agreement, which indicates nearly equal survival rates. The CRT survival rate is obviously superior to that of RT and CT alone, indicated by the other two curves. Further analysis was performed to identify the effects of other factors in Figure 3. Patients were divided into four subgroups based on age and histology. In addition to the differences between RT and CT curves in a younger age (<65), the CRT survival rate is still obviously superior to that with RT or CT. Given the possible effects of better general condition, RT is slightly better than CT in the younger age subgroup; however, neither can match the advantages of CRT.

**FIGURE 4:**
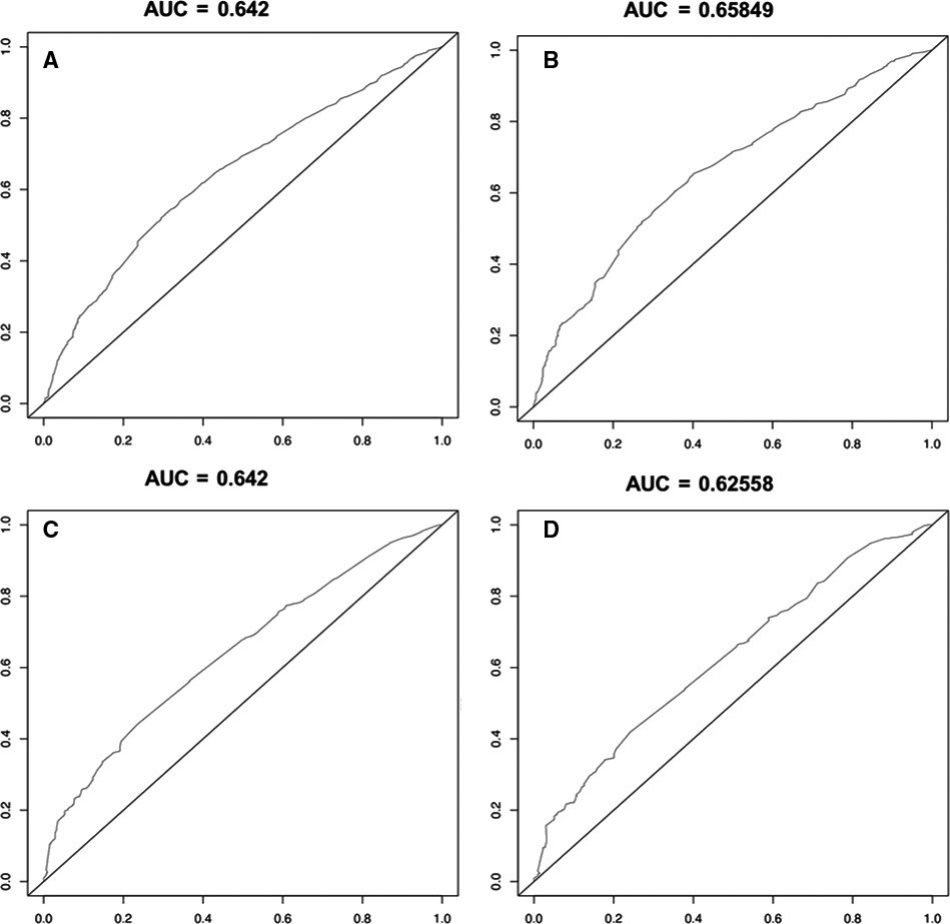
ROC curve estimating accuracy of 3‐ (A)and 5‐y (B) in the training cohort, and of 3‐(C) and 5‐y (D) in the verification cohort.

Finally, we used ROC curves to estimate the accuracy of the whole study (Figure 4). The AUC values of the 3‐ and 5‐year survival rate curves in the training cohort were 0.642 and 0.658, respectively. In the verification cohort, the values were 0.642 and 0.626.

## 4 DISCUSSION

With the increasing number of EC patients, the probability of early EC detection is increasing, especially in Asia. Although endoscopic mucosal resection and esophagectomy are the standard methods for early detection of EC, there are patients who cannot undergo this treatment. RT and CRT are the options for these patients. Due to a lack of effective assessment of the prognosis of nonoperative EC patients who receive RT or CRT, doctors have difficulty in providing the best treatment for nonoperative early EC patients. This is the problem that our study aimed to solve.

In this study, we found large sample size differences in these subgroups. The number of patients receiving CRT was far greater than that of patients receiving RT or CT (Table 1). It can be estimated that CRT patients account for a large proportion of all patients after 2015. Data for patients in this study were collected over a period of 12 years. During the time, there have been advancements in medicine, such as RT technology and support treatment. We have observed that the OS does improve. We found that the survival of patients receiving CRT was better than that of patients receiving CT and RT, especially in younger females. We divided the sample into different subgroups, including <65 and ≥65 subgroups, squamous carcinoma and adenocarcinoma subgroups, and stage I/IIA/IIB subgroups. The outcomes of each subgroup show that CRT led to a better prognosis. Considering the high incidence of EC, mostly squamous cell carcinoma, in Asia,^9^ these results provide a reference for treatment in the Asian region. Significantly, the C‐index and AUC values are not ideal; thus, they may make the accuracy doubtful.

Most of the existing studies have focused on adjuvant treatment of surgical patients, and the majority are stage III‐IV patients.^10^, ^11^, ^12^ For early stage patients, the studies were mainly related to the comparison between ET and esophagectomy,^13^, ^14^ different CT regimens, ^6^, ^15^, ^16^ or different external irradiation doses.^17^, ^18^ A study indicated that RT was not appropriate for the treatment of elderly patients with early EC.^13^ The conclusion supports our results that RT OS is as poor as CT OS in the elderly age subgroup. A recent study pointed out that concurrent CRT in elderly patients (a total of 185) shows no significant benefit over RT alone in terms of OS and CSS.^19^ In that study, elderly patients were referred to as those who were over 80 years old. This conclusion opposed our points to some degree, but the elderly patients in our study were over 65 years old. Some of the patients over 80 years old were unable to complete the planned RT. Advanced age and limited sample size could have led to biased conclusions. CRT for patients over 80 years old needs further research. Another study showed that heart and lung toxicity of RT and poor tolerance contributed to a lower rate of RT in elderly patients.^20^ In our study, RT was also an option that was not often selected. Regrettably, the SEER database could not provide an evaluation of patients’ conditions, such as performance status and comorbidity information. One report referred to a conclusion similar to ours that patients who underwent definitive CRT for EC in stage I/II showed good prognosis,^7^ but it should be further noted that the sample number was just 25.

In theory, early EC without lymph node metastasis can be regarded as a local lesion. RT alone will bring ideal effects and minor side effects. However, the results of this study surprise us. CRT has a better OS. Some studies can interpret this tendency. A previous study showed that micrometastasis frequently occurred in lymph nodes in EC patients with N0 after conventional histopathology.^21^ There was a negative tendency of positive micrometastasis on OS in EC patients. Radiation plus CT could theoretically improve local control of micrometastasis. Meanwhile, CT not only provided systematic therapeutic effects for tumor control but also enhanced the effects of RT as a radio‐sensitizing agent.^22^ This result guides us to recommendations for effective and adequate treatment for early EC.

Although we conclude that CRT is more effective than radio‐ or CT alone based on a large population sample of 5332 patients, what cannot be ignored is that limitations exist in the use of the SEER database. First, this is a retrospective study. Patients lacked homogeneity. Some patients might have underlying diseases, thus influencing OS with cancer. Next, we just know about whether patients received RT or CT. Other specific information was blinded to us, for instance, radiation dose and frequency, CT regimens and drug dose, and sequential or synchronous therapy. However, in the current study, we did not carry out quantitative analysis. The problems mentioned above need further research.

## 5 CONCLUSION

In conclusion, CRT results in better OS and CSS than CT or RT alone in nonoperative early stage patients.

## CONFLICT OF INTERESTS

The authors declare that there is no conflict of interests regarding the publication of this article.

## AUTHOR CONTRIBUTIONS

YC and JW contributed to the conception of the study; JW and JL contributed significantly to analysis and manuscript preparation; JL performed the data analyses and wrote the manuscript; YJ reviewed the manuscript and gave valuable suggestions.

## Data Availability

The data that support the findings of this study are available from the corresponding author upon reasonable request.
